# Dual Role of a Viral Polymerase in Viral Genome Replication and Particle Self-Assembly

**DOI:** 10.1128/mBio.01242-18

**Published:** 2018-10-02

**Authors:** Xiaoyu Sun, Serban L. Ilca, Juha T. Huiskonen, Minna M. Poranen

**Affiliations:** a Molecular and Integrative Biosciences Research Programme, Faculty of Biological and Environmental Sciences, University of Helsinki, Helsinki, Finland; b Division of Structural Biology, Wellcome Centre for Human Genetics, University of Oxford, Oxford, United Kingdom; c Helsinki Institute of Life Science HiLIFE, University of Helsinki, Helsinki, Finland; Rutgers, The State University of New Jersey; National Institutes of Health

**Keywords:** *Pseudomonas* phage phi6, RNA-dependent RNA polymerase, bacteriophage assembly, cystovirus, double-stranded RNA virus, virus assembly

## Abstract

Double-stranded RNA (dsRNA) viruses package several RNA-dependent RNA polymerases (RdRp) together with their dsRNA genome into an icosahedral protein capsid known as the polymerase complex. This structure is highly conserved among dsRNA viruses but is not found in any other virus group. RdRp subunits typically interact directly with the main capsid proteins, close to the 5-fold symmetric axes, and perform viral genome replication and transcription within the icosahedral protein shell. In this study, we utilized *Pseudomonas* phage Φ6, a well-established virus self-assembly model, to probe the potential roles of the RdRp in dsRNA virus assembly. We demonstrated that Φ6 RdRp accelerates the polymerase complex self-assembly process and contributes to its conformational stability and integrity. We highlight the role of specific amino acid residues on the surface of the RdRp in its incorporation during the self-assembly reaction. Substitutions of these residues reduce RdRp incorporation into the polymerase complex during the self-assembly reaction. Furthermore, we determined that the overall transcription efficiency of the Φ6 polymerase complex increased when the number of RdRp subunits exceeded the number of genome segments. These results suggest a mechanism for RdRp recruitment in the polymerase complex and highlight its novel role in virion assembly, in addition to the canonical RNA transcription and replication functions.

## Abstract (precis)

Double-stranded RNA viruses infect a wide spectrum of hosts, including animals, plants, fungi, and bacteria. Yet genome replication mechanisms of these viruses are conserved. During the infection cycle, a proteinaceous capsid, the polymerase complex, is formed. An essential component of this capsid is the viral RNA polymerase that replicates and transcribes the enclosed viral genome. The polymerase complex structure is well characterized for many double-stranded RNA viruses. However, much less is known about the hierarchical molecular interactions that take place in building up such complexes. Using the bacteriophage Φ6 self-assembly system, we obtained novel insights into the processes that mediate polymerase subunit incorporation into the polymerase complex for generation of functional structures. The results presented pave the way for the exploitation and engineering of viral self-assembly processes for biomedical and synthetic biology applications. An understanding of viral assembly processes at the molecular level may also facilitate the development of antivirals that target viral capsid assembly.

## INTRODUCTION

Viruses with double-stranded RNA (dsRNA) genomes infect a wide range of hosts, including bacteria (members of the family *Cystoviridae*), simple eukaryotes (e.g., members of the families *Totiviridae* and *Partitiviridae*), humans, and economically important livestock animals and crop plants (members of the family *Reoviridae*). Regardless of the host, most dsRNA viruses share similar genome replication strategies whereby the genome is amplified and expressed within an elaborate protein nanocompartment called the polymerase complex (1, 2). The polymerase complex of dsRNA viruses consists of 120 copies of the major capsid protein arranged as 60 asymmetric dimers to form a *T*=1 icosahedral lattice (2, 3). Packaged within the complex are several copies of the virus-encoded RNA-dependent RNA polymerase (RdRp), which is the key enzymatic component of the complex. The numbers of the RdRp subunits differ depending on the virus and typically follow the number of genome segments (4–8). *Pseudomonas* phage Φ6 represents an exception as it has approximately three to four RdRp subunits per each of the three genome segments (9). Regardless of the virus, the RdRp subunit typically interacts directly with the major capsid protein of the polymerase complex on or near the 5-fold axes of icosahedral symmetry inside the particle (5–7, 10, 11).

Although the architecture of the virion shell is well characterized for many dsRNA viruses, the molecular details of the assembly pathways, particularly the mechanisms that secure RdRp incorporation in the inner capsid shell, are not well understood for many dsRNA viruses. This process is probably best described for totiviruses (e.g., Saccharomyces cerevisiae virus L-A) which produce capsid protein-RdRp fusion proteins that coassemble with the capsid proteins to form the viral capsid (12, 13). In *Pseudomonas* phage Φ8 (family *Cystoviridae*), the RdRp nucleates the polymerase complex self-assembly, ensuring that particles lacking RdRp are not formed (14). In picobirnaviruses, viral genomic single-stranded RNAs (ssRNAs) likely facilitate RdRp incorporation into the polymerase complex (15). Similarly, RNA-RNA and RNA-RdRp interactions are also important during rotavirus self-assembly (16, 17). However, rotavirus and *Pseudomonas* phage Φ6 were previously shown to self-assemble in the absence of the RdRp subunit, although RdRp-containing complexes are formed when the RdRp and the major capsid protein are coexpressed (18, 19). This shows that the RdRp can incorporate into the polymerase complex in the absence of viral RNA and raises the issue of how RdRp incorporation in the particle is ensured.

**FIG 1:**
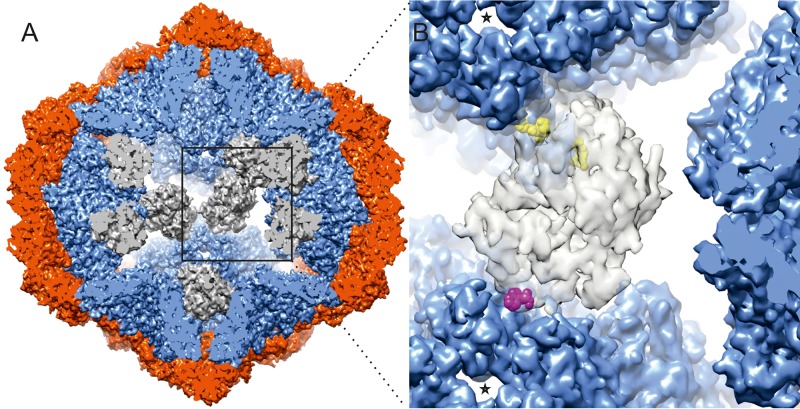
Location of the RdRp P2 within the Φ6 PC shell (EMD-3185) and P2-P1 interactions (40). (A) Eight P2 RdRps (gray) are placed on their identified binding sites within an empty PC with respect to P1A (blue) and P1B (red) subunits. (B) The orientation of P2 within the PC and its interactions with P1 residues of two neighboring 5-fold vertices (5-fold symmetr*y* axes marked by stars). The two main contact sites (His_443_ and Glu_444_; Arg_470_) on the P2 palm domain are colored in yellow and the one (Trp_576_) on the thumb domain in purple.

*Pseudomonas* phage Φ6 (family *Cystoviridae*) is among the leading model systems used to study dsRNA virus self-assembly and genome replication processes (2, 20, 21). During virion formation, an empty polymerase complex (procapsid [PC]) is first assembled. Subsequently, the PC specifically recognizes and sequentially packages the three viral genomic ssRNA precursor molecules in the order small, medium, and large (*s*, *m*, and *l*, respectively) (22, 23). The packaging of the *l* segment stimulates the synthesis of the dsRNA genome (S, M, and L), which serves as a template for plus-strand synthesis to produce viral mRNAs (24, 25). During genome packaging and replication, the polymerase complex undergoes conformational rearrangements from a compact dodecahedron with convex faces to an expanded sphere, resulting in a 2.4-fold increase of the interior volume (26, 27). The dodecahedral framework of the Φ6 polymerase complex is composed of the main structural P1 protein arranged as 60 asymmetric P1A/P1B dimers (26). The P1A subunits are arranged as pentamers around the 5-fold symmetr*y* axes and are oriented inward. The P1B subunits around the 2-fold and 3-fold axes of symmetry connect these P1A pentamers (26, 28) (Fig. 1A). Twelve copies of hexameric packaging NTPase P4 are located at the 5-fold vertices on the PC surface. P4 is essential for the nucleation of PC self-assembly, stabilizes the conformation of the empty PC, and guides viral ssRNA molecules in and out of the particle (20, 29–33). Minor protein P7 accelerates PC self-assembly (20, 34) and is located at positions around the 3-fold symmetr*y* axes of the PC interior (35, 36). The RdRp P2 is localized at the 3-fold axes with 20 potential binding sites in the PC (11, 37) (Fig. 1A). Nevertheless, only about half of these binding sites are occupied in the Φ6 virions (9, 38). When and how the P2 subunits are incorporated into Φ6 PC remain unclear.

Here, we assessed the potential role of Φ6 RdRp in PC formation in the self-assembly system and analyzed the replication and transcription activities of the self-assembled particles. The results indicate that the RdRp P2 accelerates the Φ6 self-assembly process and stabilizes the compact conformation of the P1 shell. This is likely due to specific interactions between P2 and P1 in the compact state of the particle (11) (Fig. 1B). Three different P2 surface mutants were designed to further probe the role of the specific P1-P2 interfaces. Normal incorporation of these modified RdRps during the PC self-assembly was compromised. The self-assembly system also allowed us to produce Φ6 PCs with different amounts of the RdRp subunit. Particles containing only a few RdRps were capable of genome replication. However, optimal transcription efficiency required that the average copy number of P2 was close to that observed in the Φ6 virions and that additional P2’s did not further boost the RNA synthesis activity. Taken together, our results show that P2 has a role not only in RNA replication and transcription but also in PC assembly, which provides a rationale for the incorporation of its mechanism into the PC.

## RESULTS

### RdRp subunits contribute to the sedimentation velocity of self-assembled Φ6 P1P4 particles.

**FIG 2:**
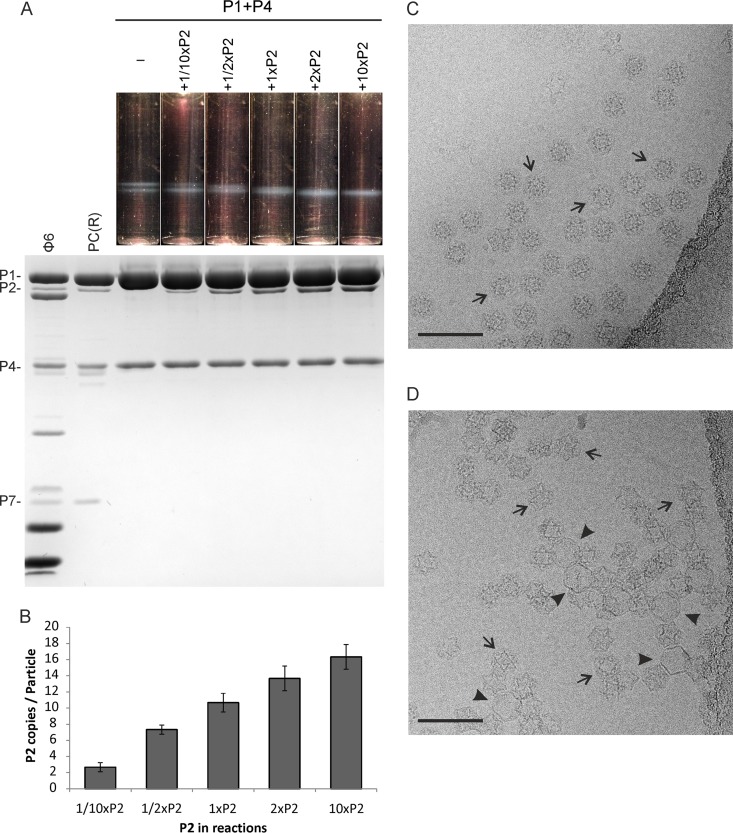
Dose-dependent incorporation of the P2 RdRp subunits into the Φ6 P1P4 particles during the self-assembly reaction is reflected in the sedimentation properties and morphology of the particles. (A) *In vitro* assembly assays were performed with increasing amount of P2 subunits. “1×P2” refers to a molar ratio of 12:120 between P2 and the main structural protein P1. The reaction products were analyzed by rate-zonal centrifugation using a linear 10% to 30% (wt/vol) sucrose gradient. The light-scattering zones of the gradients (upper panel) were collected and analyzed by SDS-PAGE. Recombinant PCs [PC(R)] from E. coli and purified Φ6 virions were used as protein size markers. The PC proteins are indicated on the left. (B) The calculated relative copy numbers of P2 in the self-assembled P1P2P4 particles are shown with bar graphs. Error bars represent standard deviations of the means of results from three repetitions. (C and D) Cryo-EM micrographs of self-assembled P1P210_×_P4 particles originating from the 10×P2 reaction (C) and P1P4 particles (D). Expanded particles are indicated with arrowheads and compact particles with arrows. Bar, 100 nm.

Previous studies have shown that P7 accelerates the formation of the complete Φ6 PC and of particles that lack P2 (P1P4 particles) (20). However, whether there is any possible effect of the P2 RdRp in the Φ6 PC self-assembly reaction has remained unclear. More-recent structural studies have proposed that P2 and P7 binding sites overlap within the empty PC (35, 36). To specifically analyze the potential role of P2 in the assembly of the Φ6 PC, we set up *in vitro* self-assembly reactions of P1P4 and P1P2P4 particles without P7. We used a fixed molecular ratio of P1 to P4 (120:72, corresponding to 12 P4 hexamers per P1 shell), no P7, and various amounts of P2. A 120:12 molar ratio of P1 to P2 (in a reaction to produce P1P2_1x_P4 particles [denoted “1×P2” here]) closely matches the stoichiometry observed in Φ6 virions (9) (Table 1). The titers representing the amounts of P2 in the self-assembly reactions were determined and showed that the amounts ranged from no P2 at all to 10×P2 (Table 1), and the resulting self-assembly products (P1P4, P1P2_1/10x_P4, P1P2_1/2x_P4, P1P2_1x_P4, P1P2_2x_P4, and P1P2_10x_P4 particles) were analyzed by rate-zonal centrifugation (Fig. 2A).

**TABLE 1:** Molar ratio of P1 to P2 in self-assembly reactions

Particle type	Name of the self- assembly reaction	Molar ratio of P1:P2
P1P4	P1P4 reaction	120:0
P1P2_1/10x_P4	1/10×P2 reaction	120:1
P1P2_1/2x_P4	1/2×P2 reaction	120:2
P1P2_1x_P4	1×P2 reaction	120:12
P1P2_2x_P4	2×P2 reaction	120:24
P1P2_10x_P4	10×P2 reaction	120:120

From the sedimentation profiles, we observed that the self-assembled P1P4 particles that lacked P2 produced two light-scattering zones, or bands, of equal intensities (Fig. 2A). Adding P2 in increasingly larger amounts to the self-assembly reactions (from 1/10×P2 to 10×P2) led to an increasingly stronger lower band and weaker upper band, to the extent that only the lower band was observed in the resulting P1P2_2x_P4 and P1P2_10x_P4 particles (Fig. 2A). The region corresponding to the two bands was collected and analyzed by sodium dodecyl sulfate-polyacrylamide gel electrophoresis (SDS-PAGE) to estimate the relative P2 copy numbers in the self-assembly products (Fig. 2B) as described previously (9). The relative P2 copy number appeared to be highly dependent on the P2 amount used in the reactions (Fig. 2B), which is consistent with previous self-assembly studies performed in the presence of P7 (9). The self-assembled P1P2_10x_P4 particles contained up to approximately 17 copies of P2, whereas the copy number of P2 in the P1P2_1x_P4 particles was close to that observed in the Φ6 virions (Fig. 2B) (9). Regardless of the P2 content, all the self-assembled particles contained similar amounts of P4 (Fig. 2A), which excludes the possibility that the packaging NTPase P4 contributes to the observed differences in sedimentation profiles. Hence, the differences in the sedimentation profiles could be directly attributed to the differing amounts of P2 in the particles (Fig. 2A and B).

### The RdRp subunit stabilizes the compact conformation of the Φ6 P1 shell.

To understand the origins of the rapidly and slowly sedimenting self-assembly products and the effects induced by P2, we analyzed the two light-scattering bands originating from the P1P4 self-assembly reaction (Fig. 2A and the leftmost sedimentation profile) in more detail. These two bands represent two different P1P4 particle populations that have distinct sedimentation properties. Such differences can arise from either particle collapse or particle expansion (resulting in changes in the particle volume or the frictional coefficient) or from differences in protein composition (resulting in changes in particle mass or density) or both. The two subpopulations of P1P4 particles had similar protein compositions (see Fig. S1 in the supplemental material), which excludes the possibility that the observed difference in the sedimentation velocity arose from mass differences and suggests that the two bands represent different conformations of the particle.

10.1128/mBio.01242-18.1 FIG S1 Subpopulation analysis of self-assembled P1P4 particles. An *in vitro* P1P4 assembly reaction was analyzed by rate-zonal centrifugation using a linear 10% to 30% (wt/vol) sucrose gradient. The area covering the light-scattering zones (indicated by the red square bracket) was continually collected into 100-µl fractions using a BioComp gradient fractionator and analyzed by SDS-PAGE. Purified Φ6 virions were used as a protein size marker; the PC proteins are indicated on the left. The quantified P1 intensities of each fraction are plotted above the gel, showing the separation of the two light-scattering bands. The relative number of P4 per P1 shell in each fraction is indicated at the bottom. Download FIG S1, PDF file, 1.9 MB. Copyright © 2018 Sun et al. 2018 Sun et al. This content is distributed under the terms of the Creative Commons Attribution 4.0 International license.

The conformational states of the P1P2_10x_P4 and P1P4 particles were further analyzed by cryogenic electron microscopy (cryo-EM). The cryo-EM micrographs of the self-assembled P1P2_10x_P4 particles originating from the 10×P2 reaction displayed a monodisperse field of compact particles (Fig. 2C). In the case of P1P4 particles, we observed both compact and expanded conformations (Fig. 2D) that were similar to those detected previously in preparations of recombinant P1P4 particles (39). In the observed cryo-EM images, the proportions of the two conformations were not equal. The ratio of intact expanded particles to compact particles was less than 1:100, and intact expanded P1P4 particles could be found only in aggregates of particles. We cannot exclude the possibility that most of the expanded P1P4 particles were in aggregates too large to be visualized by this method. The expanded and compact particles likely represent the slowly and rapidly sedimenting P1P4 particle populations, respectively. Furthermore, P1P4 preparations showed partially dissociated particles (Fig. 2D), whereas P1P2_10x_P4 particles did not, suggesting that P2 contributes to particle stability.

To further study the compact conformation of the P1P4 particle, its structure was determined at 4.8-Å resolution by cryo-EM and single-particle analysis. The structure of the P1 shell is virtually indistinguishable from that of the P1P2_10x_P4 particles (Fig. S2) (11). The main differential density arose from the absence of the RdRp in the P1P4 structure. Taken together, these data indicate that P2 stabilizes the compact form of the P1 shell, which is prone to expansion or dissociation in the absence of P2.

10.1128/mBio.01242-18.2 FIG S2 Comparison between the structure of P1P4 determined here and the previously published structure of P1P2P4 (both at 4.8Å resolution). P1P2P4 (left), P1P4 (middle), and the difference map (right) were rendered as the isosurface representation of full particles (A), a central slab in 3D (B), and a central slab in 2D (C). In panels A and B, noise regions smaller than 30 voxels were manually removed for visualization purposes using the “Hide Dust” function in UCSF Chimera (Goddart et al., 2007, J Struct Biol, 157:281-287). For panel C, the grayscale values were modified for uniformity. Bar for all panels, 10 nm. Download FIG S2, PDF file, 0.6 MB. Copyright © 2018 Sun et al. 2018 Sun et al. This content is distributed under the terms of the Creative Commons Attribution 4.0 International license.

### The number of RdRp subunits in the Φ6 PCs.

**FIG 3:**
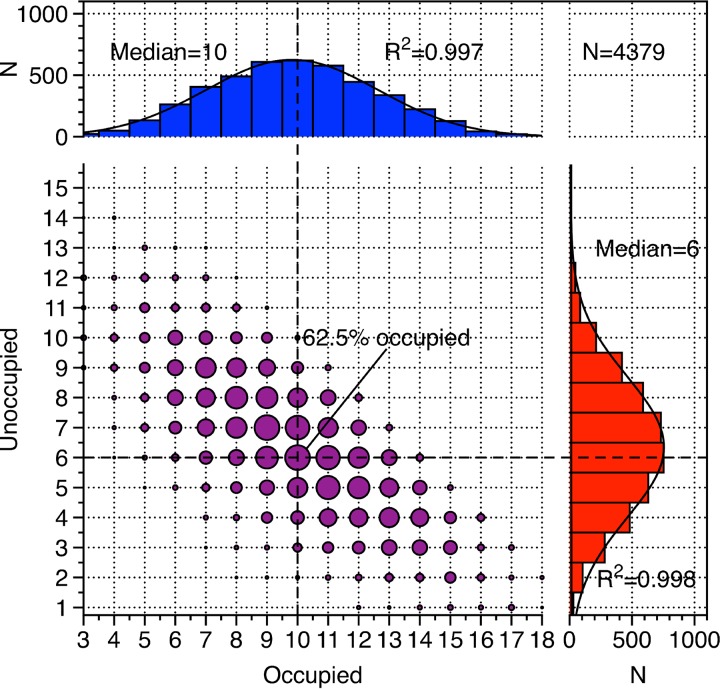
P2 copy number in the self-assembled compact P1P2_10x_P4 particles. The 20 potential binding sites per particle were classified, and three types were discerned: occupied (*x* axis), unoccupied (*y* axis), and unresolved (remaining data). The points (purple) represent each combination of the number of occupied versus unoccupied sites. The area of the points corresponds to the number of particles with the given combination in the data set. The blue histogram shows the number of sites with P2, and the red histograms shows the number of sites without P2. The medians of both histograms are indicated, and the total number of sites analyzed (N) is given.

Our biochemical analysis of P1P2_10x_P4 particles produced in the presence of excess P2 suggested that they had 17 P2’s on average at the ensemble level (Fig. 2B). However, this analysis does not address the issue of whether each particle has the same amount of P2 or, for example, if there are some particles that have none and some that have a full complement of P2’s. To quantify the number of P2’s on a particle-by-particle basis, we computationally analyzed the presence and absence of P2 in the 20 possible binding sites for each particle cryo-EM image (11, 40). The P2 copy number followed a broad binomial distribution, with the particles containing on average 10 discernible positions with P2 and 6 without P2, while the rest of the positions could not be classified (Fig. 3). On average, P2 thus occupies 62.5% of the positions, corresponding to 12 to 13 P2’s. Similar broad binomial distributions were observed earlier in Φ6 PCs, where 8 P2’s on average were estimated to bind, consistent with a random incorporation model (41). However, we cannot exclude the possibility that the broad nature of the distribution is at least partially due to the relatively low signal-to-noise ratio in the cryo-EM images and that there is one population of particles in which all of the particles contain roughly the same amount of P2’s. It is also possible that some P2’s could be incorporated unspecifically inside of the particles (42); such a population of P2’s would appear as disordered density in our cryo-EM maps and be undetectable by this analysis.

### P2 accelerates PC self-assembly in the absence of P7.

**FIG 4:**
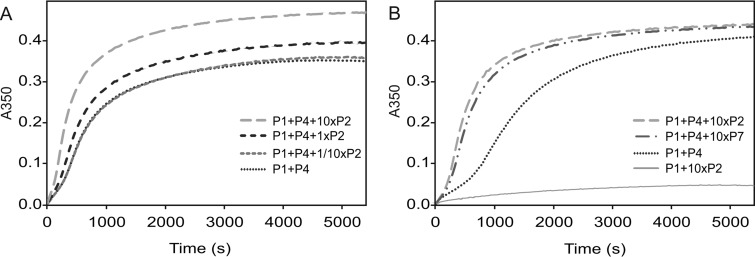
Increase in light scattering during the *in vitro* assembly reaction of the Φ6 P1 shell. (A) Effect of P2 RdRp titration on the self-assembly kinetics of the P1P2P4 particle. (B) Comparison of the effects of P2, P4, and P7 on the assembly kinetics of the P1 shell. The molar ratio of a 1× reaction represents 12:120 for P2:P1 and 45:120 for P7:P1.

The effects of P2 on PC stability and conformation (Fig. 2) suggested that the RdRp subunit of Φ6 could also have a role in the self-assembly of the empty PC. This hypothesis was tested by analyzing the effect of P2 on the kinetics of the Φ6 PC self-assembly reaction in the absence of the P7 assembly cofactor. In general, P2 increased the P1P4 assembly rate in a dose-dependent manner (Fig. 4A), an effect similar to that observed for P7 (Fig. 4B) (9, 20, 34). The final level of light scattering was approximately 10% to 30% higher in the 10×P2 reaction than in the other reactions (Fig. 4; total of 10 repetitions), suggesting that the overall yield of particles was increased in the presence of P2. This observation further supports the concept that P2 contributes to the stability of the Φ6 PCs.

Taken together, our observations indicate that Φ6 P2 may have a more important role in the PC self-assembly process than previously anticipated. To test the possibility that P2 could be used instead of P4 in Φ6 PC nucleation (as in the case of *Pseudomonas* phage Φ8) (14), we performed a Φ6 self-assembly reaction using equimolar amounts of the P1 major capsid protein and RdRp P2 (10×P2 reaction). The assembly reaction showed little visible change in light scattering (Fig. 4B), and most of the protein subunits were retained in the top fraction after sedimentation (data not shown). Thus, Φ6 P2, even if present in 10-fold molar excess with respect to the amount found in the final assembly product, cannot replace P4 in the nucleation of P1 shell assembly. This observation is consistent with our recent observation that the P4 C-terminus may be important in nucleating the P1 shell assembly in Φ6 (33).

### The transcription efficiency of Φ6 PC is dependent on the amount of the P2 RdRp in the particles.

**FIG 5:**
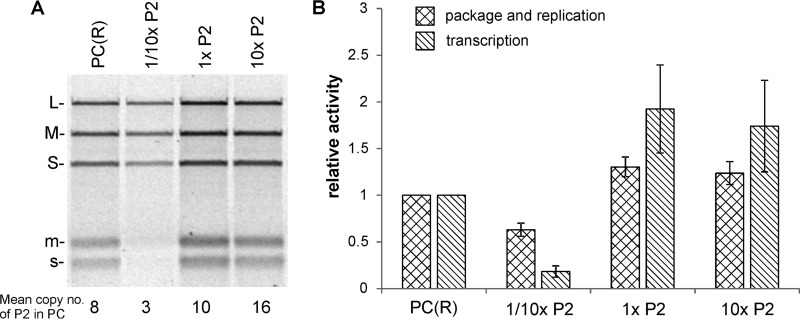
Effect of RdRp copy number on the minus-strand and plus-strand synthesis activities of self-assembled Φ6 PCs. Plus-strand synthesis reactions (combined *in vitro* ssRNA packaging, replication, and transcription reactions) were performed with self-assembled PCs containing different amounts of P2 RdRp. (A) Agarose gel electrophoresis analysis of the reaction products. Positions of double-stranded (uppercase letters) and single-stranded (lowercase letters) RNA segments are indicated on the left. Average copy numbers of P2 subunits in the different PC preparations are shown at the bottom. (B) The relative replication and transcription activity levels were calculated based on phosphorimage quantitation of labeled RNA molecules and are shown in bar graphs. Error bars represent standard deviations of the means of results from three repetitions.

The Φ6 *in vitro* self-assembly system allowed us to produce Φ6 PC containing different amounts of RdRp subunits (Fig. 2A and B) and to investigate the effect of the RdRp copy number on the enzymatic activities of the PC. We produced self-assembled Φ6 PCs containing mean copy numbers of 3, 10, and 16 P2’s per particle (originating from 1/10×P2, 1×P2, and 10×P2 reactions, respectively; Fig. 5A). All the particles used for the activity analyses were processed in the presence of P7 because P7 is required for normal RNA synthesis activity (18, 34, 43). The amounts of P7 were equal in the particles produced. The enzymatic activity of the freshly prepared particles was analyzed using Φ6 *in vitro* ssRNA packaging, replication, and transcription analyses. The activity was compared to that seen with PCs produced using a recombinant Escherichia coli expression system. Under the applied reaction conditions, the Φ6-specific ssRNA templates (*s*, *m*, and *l*) were packaged into the empty PCs and converted to dsRNA molecules (S, M, and L), which subsequently served as templates for the synthesis of *m* and *s* transcripts (the late-transcription mode) via a semiconservative strand displacement mechanism (23, 25, 44). The reactions were performed in the presence of radioactively labeled UTP and resulted in the incorporation of the label in the negative and positive strands of the dsRNA and in the ssRNA transcripts.

The PCs that were assembled *in vitro* with a mean copy number of 10 P2’s were enzymatically slightly more active than similar recombinant particles produced in E. coli (Fig. 5A, lanes 1 and 3), which is consistent with our previous studies (20). Regardless of the overall activity of the particles, the relative levels of replication efficiency of the three genomic RNAs did not vary. This suggests that the particles could package the three genomic ssRNA molecules with equal levels of efficiency; that is, the differences in P2 copy number did not interfere with the normal packaging regulation (Fig. 5A).

The PCs containing three P2’s on average displayed a reduced level of RNA synthesis compared to the recombinant PCs (Fig. 5A and lane 2). The measured signal intensity of the produced dsRNA was approximately 50% of the corresponding intensities measured for the recombinant PCs containing approximately eight P2’s per particle, and that of ssRNA was about 10% to 20% (Fig. 5B). The lower level of general RNA synthesis activity of these particles could be an indication of unequal RdRp distributions within the particle population and of the presence of an inactive subpopulation that lacks RdRps. Nevertheless, the transcriptional activity of the active population of particles was clearly compromised (low ssRNA-to-dsRNA ratio) if the RdRp copy number was below that observed in the virions.

The particles originating from the 10×P2 assembly reactions showed packaging and replication activities similar to the activities seen with those from the 1×P2 reaction (Fig. 5B), which indicates that the redundant RNA polymerases are unable to boost the production of transcripts. The RdRp copy number observed in the Φ6 virions (approximately 3 to 4 copies per genome segment) is thus optimal for viral RNA synthesis.

### Amino acid substitutions at the predicted P2-P1 interaction sites reduce P2 incorporation into the P1 shell.

What are the molecular interactions that ensure the incorporation of Φ6 RdRp in the assembling PC? Our recent structural analyses of P1P2P4 particles indicated that P2 RdRp interacts with the main structural P1 protein through two distinct contact sites (11). One contact surface interacts with P1 through salt bridges involving residues Arg_470_, His_443_, and Glu_444_ residing in the palm domain of P2, while the other is localized on the fingers domain and involves a hydrophobic interaction through Trp_576_ (Fig. 1B). To validate these predicted interaction sites, we designed three P2 surface mutants, namely, HER (His_443_ to Ala, Glu_444_ to Gln, and Arg_470_ to Ala), W576A (Trp_576_ to Ala), and HERW (all substitutions; see Table S1 in the supplemental material). The purified histidine-tagged RdRp variants catalyzed dsRNA synthesis *in vitro* with activity similar to that seen with the histidine-tagged P2 (His-P2), used here as a control (Fig. S3). This confirms that the amino acid substitutions in HER, W576A, and HERW did not interfere with the normal folding of the proteins.

10.1128/mBio.01242-18.3 FIG S3 Agarose gel analysis of replication reaction products synthesized by His-P2 and different P2 surface mutants. Minus-strand synthesis reactions were performed using Φ6 S-segment-specific plus-strand ssRNA (*s*) as a template. Positions of double-stranded (uppercase letters, products) and single-stranded (lowercase letters, template) RNA are indicated on the left. Download FIG S3, PDF file, 0.3 MB. Copyright © 2018 Sun et al. 2018 Sun et al. This content is distributed under the terms of the Creative Commons Attribution 4.0 International license.

10.1128/mBio.01242-18.5 TABLE S1 Φ6 RdRp surface mutants and oligonucleotides used for site-directed mutagenesis. Download Table S1, PDF file, 0.0 MB. Copyright © 2018 Sun et al. 2018 Sun et al. This content is distributed under the terms of the Creative Commons Attribution 4.0 International license.

**FIG 6:**
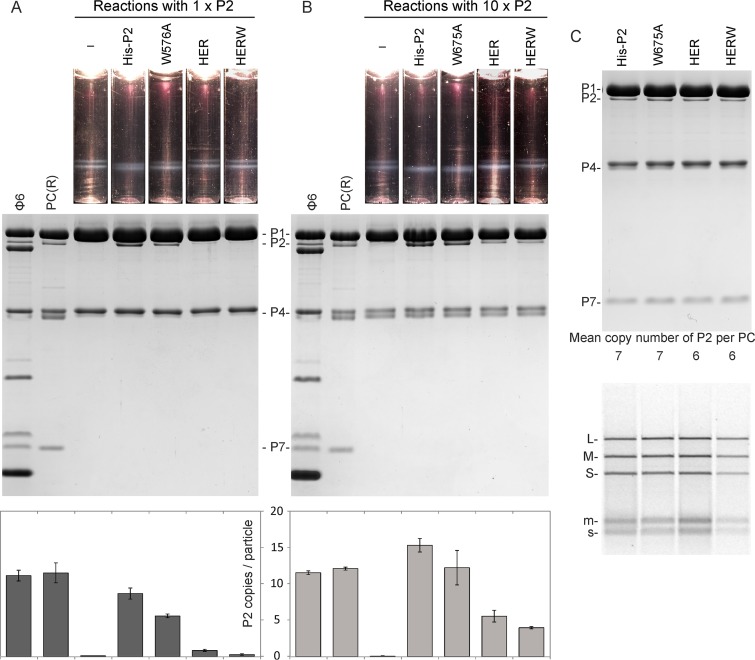
Incorporation of P2 surface mutants into P1P4 particles and the enzymatic activities of the particles. (A and B) *In vitro* assembly assays were performed using a molar ratio of 12:120 between the P2 surface mutants and the main structural protein P1 (1 × P2) (A) or a 10-fold excess of P2 (10 × P2) (B). The reaction products were analyzed by rate-zonal centrifugation using a linear 10% to 30% (wt/vol) sucrose gradient. The light-scattering zones were collected and analyzed by SDS-PAGE. Recombinant PCs [PC(R)] from E. coli and purified Φ6 virions were used as protein size markers. The PC proteins are indicated in the middle of the SDS-PAGE gels. The relative copy numbers of P2 in the P1P2P4 particles are shown with bar graphs. Error bars represent standard deviations of the means of results from three repetitions. (C) SDS-PAGE analysis of self-assembled PCs with approximately equal amounts of the different P2 mutants per particle. The calculated average copy numbers for P2 subunits are shown at the bottom of the panel. The particles were used for plus-strand synthesis reactions (lower panel). The products were analyzed using agarose gel electrophoresis. The positions of double-stranded (uppercase letters) and single-stranded (lowercase letters) RNA segments are indicated on the left.

To analyze the impact of the amino acid substitutions on the incorporation of P2 into the P1 shell, we set up P1P4 self-assembly reactions with the different P2 surface mutants and compared them to His-P2 results. The histidine tag in P2 showed no effect with respect to its incorporation efficiency (Fig. S4). However, all the P2 surface mutants displayed reduced incorporation efficiencies during the PC self-assembly reaction (Fig. 6A and B). After the 1×P2 assembly reactions, averages of six copies of W576A and one copy of HER and minimal HERW could be detected in the corresponding self-assembly products (Fig. 6A). To further probe the binding capacity of the P2 surface mutants, 10×P2 assembly reactions were set up. On average, 12, 6, and 4 copies of W576A, HER, and HERW, respectively, were incorporated into the P1P2P4 particles under these conditions (Fig. 6B). The PCs that self-assembled *in vitro* and contained His-P2, W576A, HER, or HERW instead of wild-type P2 in addition to proteins P1, P4 and P7 were also functional and able to encapsidate, replicate, and transcribe the viral genomic RNA molecules (L, M, and S; Fig. 6C). However, the activity of PCs containing six to seven RdRp subunits was slightly lower in HERW-containing particles than in those containing His-P2, W576A, or HER variants (Fig. 6C). In conclusion, the P1-P2 contact sites observed in the final assembly (i.e., in the empty polymerase complex) also mediate the incorporation of P2 during PC self-assembly.

10.1128/mBio.01242-18.4 FIG S4 Incorporation of histidine-tagged P2 and wild-type P2 in the Φ6 PC during the self-assembly reaction. *In vitro* assembly reactions were performed with increasing amounts of P2. The reaction products were analyzed by rate-zonal centrifugation using a linear 10% to 30% (wt/vol) sucrose gradient (upper panels). The light-scattering zones were collected and analyzed by SDS-PAGE (middle). Recombinant PCs [PC(R)] from E. coli and purified Φ6 virions were used as protein size markers. The PC proteins are indicated between the two gels. The relative copy numbers of P2 and P4 (bottom of the gels) were calculated based on the band intensities of the SDS-PAGE gels. Download FIG S4, PDF file, 0.1 MB. Copyright © 2018 Sun et al. 2018 Sun et al. This content is distributed under the terms of the Creative Commons Attribution 4.0 International license.

## DISCUSSION

RNA viruses typically replicate their genome within a closed protein shell (dsRNA viruses) or within a membrane compartment enclosing the genomic precursor molecules and the viral RdRp subunits (eukaryotic positive-strand RNA viruses). These structures are vital for virus reproduction, and their correct formation is a key process in the viral life cycle. How RNA viruses secure the incorporation of the RdRp subunit into their replication compartments is largely unknown. Using an *in vitro* assembly system, we have demonstrated that the assembly of the RdRp subunits into the polymerase complex of *Pseudomonas* phage Φ6 is kinetically favored (Fig. 4) and driven by specific interactions with the major capsid protein (Fig. 6).

Our results show that P2 can accelerate the P1P4 particle assembly (Fig. 4A), probably through stabilizing some late-assembly intermediates or the final assembly product. Furthermore, the particles containing only a few P2’s or no P2 (P1P4 particles) had a tendency toward structural changes leading to premature particle expansion and dissociation (Fig. 2). Taken together, these observations indicate that misassembled polymerase complexes containing suboptimal amounts of RdRps may disassemble, releasing P1 and P4 subunits for reuse in subsequent assembly reactions. Such flexibility in the assembly pathway allows the viral subunits to be recycled, thus favoring the formation of functional virions with an optimal number of RdRp copies and, ultimately, increasing the fitness of the virus.

The effect of P2 with respect to the kinetics of P1P4 particle assembly was similar to that observed for the P7 assembly cofactor (Fig. 4B). This specific effect of P2 was detected previously when the self-assembly reaction was studied in the presence of P7 (9), which suggests that the roles of P2 and P7 in the particle self-assembly are not equal despite the fact that they likely occupy similar sites in the PC (35, 36). Evidence of direct interactions between P2 and P7 of phage Φ12 has been obtained with solution nucleic magnetic resonance (45), but further studies are needed to reveal the specific interactions between P2 and P7 and how they contribute to the particle self-assembly, stability, and activities.

Conformational stability of the empty PC is essential for normal function (31, 32, 46). Premature expansion of the PC can compromise the genome packaging regulation, resulting in the encapsidation and replication of the M and L segments only. The results presented here indicate that P2 RdRp contributes to the stability of empty PCs in the compact conformation (Fig. 2). Within the empty PC, P2 interacts with two neighboring P1A pentamers, holding the adjacent 5-fold vertices in close proximity (Fig. 1B) (11) and stabilizing the compact conformation of the PCs. The specific amino acid residues on the P2 surface involved in the interactions with P1 were shown to be important for the success of P2 incorporation into the P1P4 shells (Fig. 6A and B). Amino acid substitutions in the palm domain that prevent the ionic interactions proved to be particularly detrimental (Fig. 1 and 6A and B; HER), which is consistent with the known role of electrostatic interactions in PC self-assembly and P2 incorporation (47). Nevertheless, the interactions holding P2 in its position in the empty PC were not essential for the activities of the polymerase complex, suggesting that these interactions do not play a major role in the expanded RNA-containing particle (Fig. 6C).

The virions of dsRNA viruses typically contain an RdRp subunit for each genome segment. Φ6 is an exception as it has on average approximately 10 RdRp subunits but only three genome segments. Our results show that the RdRp/genome segment ratio observed in the Φ6 virions and PCs (9) is optimal for viral genome replication and transcription activities (Fig. 5). On the one hand, lower RdRp content reduced the activity of the polymerase complexes, especially with respect to viral ssRNA production. Earlier EM analyses of Φ6 transcription intermediates showed multiple (up to five) transcription forks on S and M segments (48). This explains why particles that contain on average 10 RdRps have higher transcription efficiency than particles that have only one RdRp per each genome segment. On the other hand, addition of RdRps beyond the level observed in Φ6 virions did not further increase the activity of the self-assembled PCs (Fig. 5). The extra RdRps in these particles may interfere with each other due to steric hindrance or prevent the expansion of the particle or localize in the expanded transcribing particles at positions that impede their activity. The efficient incorporation of RdRps into PC is likely to be important for successful infection. Free RdRps are active and can utilize different ssRNA templates (49), which could result in the conversion of unpackaged viral ssRNA and cellular mRNAs into dsRNA. Having more binding sites for the RdRps (20 in total per particle) than need to be occupied for optimal activity (approximately 10 based on the results reported here) might be essential to secure efficient RdRp incorporation, thus ensuring that no free RdRps remain in the cytoplasm.

In conclusion, our results clarify the process of RdRp subunit incorporation into the Φ6 PC. Similar molecular processes may be applied in other viral assembly systems, and in other macromolecular self-assembly pathways in general, in which the incorporation of minor subunits occurs after nucleation. The self-assembly processes of viruses are emerging targets for antiviral drugs, and further research is required to understand the pathways leading to the assembly of functional viruses and viral replication complexes.

## MATERIALS AND METHODS

### Bacterial strains and plasmids.

E. coli strain BL21(DE3) (50) was used to produce P7 and all the P2 derivatives. P4 was expressed in E. coli HMS174 (50). P1P4 particles and complete recombinant PCs (P1P2P4P7 particles) were produced in E. coli JM109 (43, 51). Pseudomonas syringae pathovar phaseolicola HB10Y was used as a host for *Pseudomonas* phage Φ6 (21). E. coli XL1-Blue (Stratagene) was the host for plasmid propagation.

Plasmids pEM2, pJTJ7, and pEM7 encode proteins P2, P4, and P7, respectively (20, 49, 52). Plasmid pEM33 (53) encodes recombinant P2 with a C-terminal hexahistidine tag. Point mutations were introduced into the P2 coding sequence of pEM33 using a QuikChange mutagenesis kit (Stratagene) with appropriate oligonucleotides (see Table S1 in the supplemental material). The resultant pXS28, pXS29, and pXS30 plasmids contain point mutations on the P1-P2 contact areas of the P2 thumb domain (W576A), palm domain (HER), and both (HERW), respectively. P1P4 particles were produced from plasmid pLM358 (54) for P1 protein purification. Plasmids pLM687 (55), pLM656 (56), and pLM659 (57) contain the cDNA copies of the Φ6 L, M, and S segments, respectively, and were used for the production of Φ6-specific ssRNA molecules. Plasmid pLM687 was also used to produce recombinant PCs.

### Protein purification and particle isolation.

Wild-type proteins P1, P2, P4, and P7 were purified as previously described and stored at −80°C (20, 49, 58, 59). The C-terminally histidine-tagged P2’s (His-P2; wild type and mutants) were expressed and purified as described previously for N-terminally histidine-tagged P2 (60). Protein concentrations were determined by the Coomassie brilliant blue method using bovine serum albumin as a standard (61). Recombinant PCs from an *in vivo* assembly system were isolated as previously described and stored at −80°C (23). Phage Φ6 used as a protein size marker in SDS-PAGE was propagated and isolated according to the method described by Bamford et al. (62).

### *In vitro* PC and P1P2P4 assembly.

The reactions were performed at room temperature for 90 min in a reaction mixture containing 250 to 350 mM NaCl, 20 mM Tris (pH 8. 0), and 6% (wt/vol) polyethylene glycol 4000 using a P1 concentration of approximately 0.2 mg/ml as described previously (9, 20). Changes in light scattering, reflecting the kinetics of an *in vitro* PC self-assembly reaction, were recorded at 350 nm (bandwidth, 10 nm) in 5-s increments using a V560 UV light-visible light spectrophotometer (Jasco). Subsequently, self-assembled PCs or P1P2P4 particles were separated from unassembled subunits by rate-zonal centrifugation in a linear gradient consisting of 10% to 30% (wt/vol) sucrose and 20 mM Tris-HCl (pH 8.0) (Sorvall AH-650; 149,000 × *g*, 60 min, 15°C). The positions of the light-scattering zone were recorded, and the fractions were collected using a BioComp gradient fractionator. The protein compositions of the sedimentation fractions were analyzed by SDS-PAGE, and the relative copy numbers of minor PC proteins were quantified as described previously (9).

### *In vitro* ssRNA packaging, replication, and transcription assays.

The Φ6-specific plus-sense ssRNAs (*s*, *m*, and *l*) for the RNA synthesis assays were produced by *in vitro* transcription with T7 RNA polymerase using PCR-amplified templates from plasmids pLM659, pLM656, and pLM687, respectively (63). Plus-strand synthesis activity assays (combined *in vitro* ssRNA packaging, replication, and transcription reactions) for *in vitro*-assembled and recombinant PCs were performed according to van Dijk et al. (44) using [α-^33^P]UTP (PerkinElmer Inc.). The replication assays for His-P2 and its derivatives (HER, W576A, and HERW) were performed using standard replication reaction conditions optimized for isolated wild-type P2 (49). The reaction products were analyzed by electrophoresis in a 0.8% (wt/vol) agarose gel. Gels stained with ethidium bromide were imaged using a ChemiDoc Touch imaging system (Bio-Rad). Autoradiographs were recorded by a Fuji BAS-1500 phosphorimager and a BAS1500 image plate (Fujifilm). Quantitative estimation of band signal intensity was performed using AIDA image analyzer software (Raytest; Isotopenmeßgeräte GmbH).

### Cryo-EM data collection and analysis of P1P4 particles.

The cryo-EM structure of P1P2P4 has been published before (11), and the same data collection and processing protocols were employed here to obtain the P1P4 structure. Briefly, 3 µl of purified sample was applied on a glow-discharged copper grid coated with a film of holey carbon (C-flat; Protochips, Raleigh, NC). Data was collected on a 300-kV transmission electron microscope (Tecnai F30 Polara; FEI, Hillsboro, OR) equipped with an energy filter (GIF Quantum LS; Gatan, Pleasanton, CA) with a 20-eV slit width and direct detection camera (K2 Summit; Gatan). Electron counting superresolution mode with a dose rate of 6 to 8 e^-^/pixel/s was employed to acquire movies of 22 frames representing 0.2 s each. The frames were aligned to account for specimen drift using MOTIONCORR (64), and the contrast transfer function (CTF) parameters were estimated using CTFFIND3 (65). The particles were picked automatically in ETHAN (66) and manually curated using e2boxer.py in EMAN2. Particle extraction, two-dimensional (2D) and three-dimensional (3D) classifications, 3D refinement, particle polishing, and postprocessing of the density maps were performed in RELION using standard protocols (67). A difference map based on comparisons between P1P4 particles (EMD-0245) and P1P2P4 particles (EMD-3185) (40) was calculated by volume subtraction using the program *bop* in Bsoft. This was performed after scaling of the reconstruction amplitudes using the program *bampweigh* in the same package (68).

### P2 distribution in P1P2P4 particles.

Localized reconstruction has been applied previously to determine the low-resolution structure of P2 and to determine its average occupancy in the P1P2P4 particles (11). Here, we followed the same approach to estimate the per-particle P2 copy number in P1P2P4 particles. For each of the 20 P2 binding sites, we determined whether P2 was clearly present or clearly absent or not clearly present or clearly absent by 3D classification in RELION (11). The number of particles with different presence/absence ratios was plotted. P2 presence/absence histograms were also plotted, and a Gaussian function was fitted to both of the histograms.

### Accession number(s).

Cryo-EM density maps of the P1P4 particle reported in this paper has been deposited in the Electron Microscopy Data Bank under accession number EMD-0245.
