# Supplementary material for: Dual Role of a Viral Polymerase in Viral Genome Replication and Particle Self-Assembly
Source: mBio. 2018 Oct 2;9(5):e01242-18. doi: 10.1128/mBio.01242-18 (PMC6168860; doi:10.1128/mBio.01242-18)
Supplement: FIG S1 [file mbo005184089sf1.pdf]

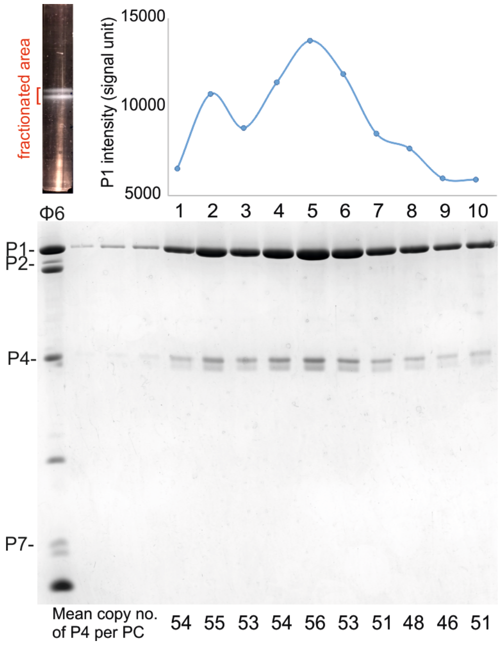

Figure S1 Subpopulation analysis of self-assembled P1P4 particles. An *in vitro* P1P4 assembly reaction was analyzed by rate-zonal centrifugation using a linear 10 to 30% (w/v) sucrose gradient. The area covering the light-scattering zones (indicated by the red square bracket) was continually collected into 100-µl fractions using BioComp gradient fractionator and analyzed by SDS-PAGE. Purified Φ6 virions were used as a protein size marker; the PC proteins are indicated on the left. The quantified P1 intensities of each fraction are plotted above the gel showing the separation of the two light-scattering bands. The relative number of P4 per P1 shell in each fraction is indicated at the bottom.
