# Supplementary material for: Dual Role of a Viral Polymerase in Viral Genome Replication and Particle Self-Assembly
Source: mBio. 2018 Oct 2;9(5):e01242-18. doi: 10.1128/mBio.01242-18 (PMC6168860; doi:10.1128/mBio.01242-18)
Supplement: FIG S2 [file mbo005184089sf2.pdf]

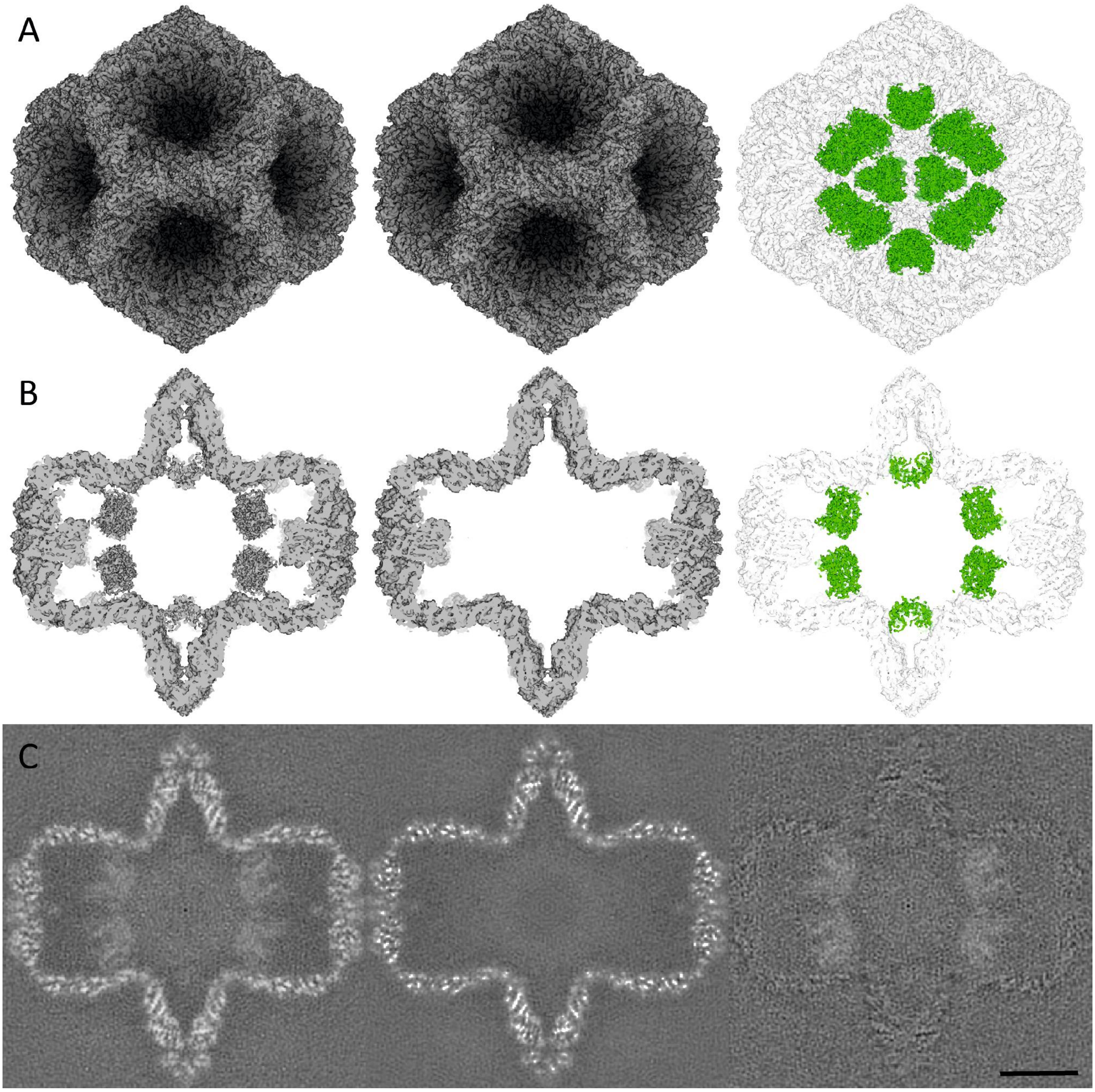


**Figure S2** Comparison between the structure of P1P4 determined here and the previously published structure of P1P2P4 (both at 4.8Å resolution). P1P2P4 (left), P1P4 (middle), and the difference map (right) were rendered as the isosurface representation of full particles (A), a central slab in 3D (B), and a central slab in 2D (C). In A and B, noise regions smaller than 30 voxels were manually removed for visualization purposes using the 'Hide Dust' function in UCSF Chimera (Goddart et al. 2007). In C, the greyscale values were modified for uniformity. Scale bar represents 10 nm for all panels.
