# Supplementary material for: Dual Role of a Viral Polymerase in Viral Genome Replication and Particle Self-Assembly
Source: mBio. 2018 Oct 2;9(5):e01242-18. doi: 10.1128/mBio.01242-18 (PMC6168860; doi:10.1128/mBio.01242-18)
Supplement: FIG S3 [file mbo005184089sf3.pdf]

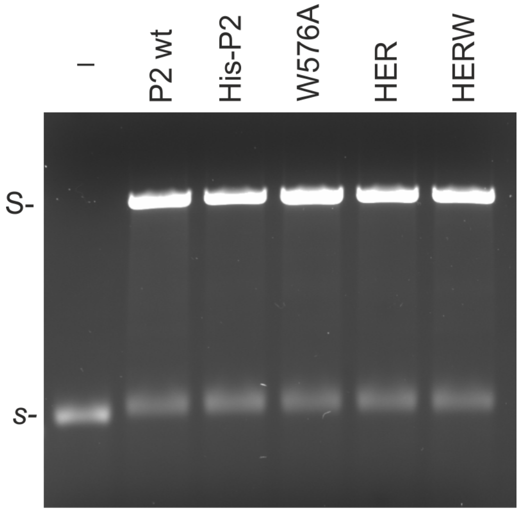

Figure S3 Agarose gel analysis of replication reaction products synthesized by His-P2 and different P2 surface mutants. Minus-strand synthesis reactions were performed using Φ6 S-segment specific plus-strand ssRNA (*s*) as a template. Positions of double-stranded (uppercase letters, products) and single-stranded (lowercase letters, template) RNA are indicated on the left.
