# Supplementary material for: Dual Role of a Viral Polymerase in Viral Genome Replication and Particle Self-Assembly
Source: mBio. 2018 Oct 2;9(5):e01242-18. doi: 10.1128/mBio.01242-18 (PMC6168860; doi:10.1128/mBio.01242-18)
Supplement: TABLE S1 [file mbo005184089st1.pdf]

Table S1: Φ6 RdRp surface mutants and oligonucleotides used for site-directed mutagenesis

RdRp	plasmid	amino acid substitution(s)	mutation site	oligonucleotide	Sequence (5' – 3') of the oligonucleotide
W576A	pXS28	W576A	thumb	W576A_FWD	GAGCGTTGCTGGGCGAACGCGTTCGGTG
				W576A_REV	CACCGAACGCGTTCGCCCAGCAACGCTC
HER	pXS29	H443A, E444Q, R470A	palm	HE4AQ_FWD	CGTATTGGCAAGGAGCCCAGGAGATCCGTCAGATCTC
				HE4AQ_RE	GAGATCTGACGGATCTCCTGGGCTCCTTGCCAATACG
				R470A_FWD	CTTTGGTTGGTGGTCATGCTTTGTTCGAGATGCTGAAAG
				R470A_RE	CTTTCAGCATCTCGAACAAAGCATGACCACCAACCAAAG
HERW	pXS30	H443A, E444Q, R470A, W576A	thumb, palm	as above	as above
