# Supplementary material for: Dual Role of a Viral Polymerase in Viral Genome Replication and Particle Self-Assembly
Source: mBio. 2018 Oct 2;9(5):e01242-18. doi: 10.1128/mBio.01242-18 (PMC6168860; doi:10.1128/mBio.01242-18)
Supplement: FIG S4 [file mbo005184089sf4.pdf]

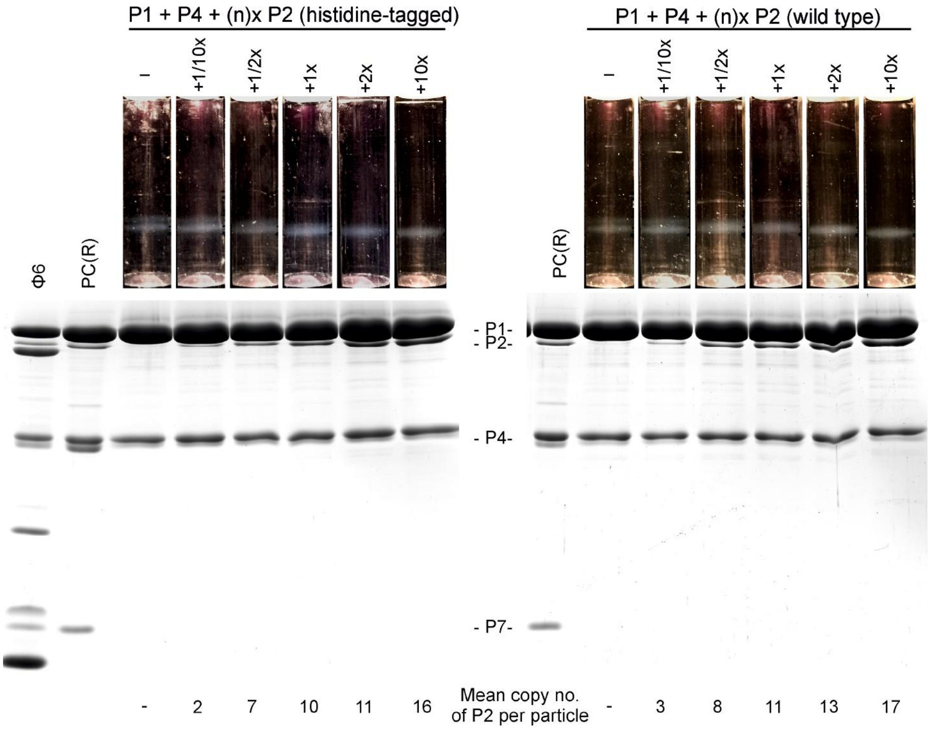

Figure S4 Incorporation of histidine-tagged P2 and wild-type P2 in the Φ6 PC during the self-assembly reaction. *In vitro* assembly reactions were performed with increasing amounts of P2. The reaction products were analyzed by rate-zonal centrifugation using a linear 10 to 30% (w/v) sucrose gradient (upper panels). The light-scattering zones were collected and analyzed by SDS-PAGE (middle). Recombinant PCs [PC(R)] from *E. coli* and purified Φ6 virions were used as protein size markers. The PC proteins are indicated between the two gels. The relative copy numbers of P2 and P4 (bottom of the gels) were calculated based on the band intensities of the SDS-PAGE gels.
